# Effective Subnetwork Topology for Synchronizing Interconnected Networks of Coupled Phase Oscillators

**DOI:** 10.3389/fncom.2018.00017

**Published:** 2018-03-28

**Authors:** Hideaki Yamamoto, Shigeru Kubota, Fabio A. Shimizu, Ayumi Hirano-Iwata, Michio Niwano

**Affiliations:** ^1^Frontier Research Institute for Interdisciplinary Sciences, Tohoku University, Sendai, Japan; ^2^Graduate School of Science and Engineering, Yamagata University, Yamagata, Japan; ^3^Research Institute of Electrical Communication, Tohoku University, Sendai, Japan; ^4^Advanced Institute for Materials Research, Tohoku University, Sendai, Japan

**Keywords:** synchronization, complex networks, modular organization, phase oscillator, Kuramoto model, synchrony alignment function

## Abstract

A system consisting of interconnected networks, or a network of networks (NoN), appears diversely in many real-world systems, including the brain. In this study, we consider NoNs consisting of heterogeneous phase oscillators and investigate how the topology of subnetworks affects the global synchrony of the network. The degree of synchrony and the effect of subnetwork topology are evaluated based on the Kuramoto order parameter and the minimum coupling strength necessary for the order parameter to exceed a threshold value, respectively. In contrast to an isolated network in which random connectivity is favorable for achieving synchrony, NoNs synchronize with weaker interconnections when the degree distribution of subnetworks is heterogeneous, suggesting the major role of the high-degree nodes. We also investigate a case in which subnetworks with different average natural frequencies are coupled to show that direct coupling of subnetworks with the largest variation is effective for synchronizing the whole system. In real-world NoNs like the brain, the balance of synchrony and asynchrony is critical for its function at various spatial resolutions. Our work provides novel insights into the topological basis of coordinated dynamics in such networks.

## Introduction

Many biological, social, and technological systems comprise of interacting subsystems and can be modeled as a network of networks (NoN) (Gao et al., 2012; Boccaletti et al., 2014; Kivelä et al., 2014). A prominent example of this is the brain that constitutes of multiple “regions” (Zamora-López et al., 2011; Bullmore and Sporns, 2012). Delving in one more spatial resolution, the neocortical region of the mammalian brain can further be regarded as an assembly of a densely connected neuronal module, called the minicolumn (Mountcastle, 1997).

In the brain, synchronized activity of neurons is essential for the development and computation. Synchronization in NoNs and modular networks has been explored theoretically based several models, including phase oscillators (Arenas et al., 2006, 2008; Barreto et al., 2008; Laing, 2009; Zhao et al., 2010; Louzada et al., 2013), chaotic oscillators (Zhao et al., 2011; Aguirre et al., 2014; Leyva et al., 2017), and various neuron models (Zhao et al., 2010; Batista et al., 2012; Prado et al., 2014), especially from the viewpoint of competition of global and local synchronizations depending on the ratio or the strength of interactions within and between the subnetworks. In a system of heterogeneous phase oscillators, Arenas et al. (2006) studied the dynamical stability of a locally synchronized state in hierarchically modular networks and provided analytical support based on the master stability function. Zhao et al. (2010) used a similar phase oscillator system to investigate the effect of modular topology on network dynamics, focusing on the relationship between the degree of topological modularization and the complexity of network dynamics. The case of globally coupled NoNs has been analytically investigated in more detail, for instance, using a self-consistent analysis (Barreto et al., 2008) or the Ott-Antonsen ansatz (Ott and Antonsen, 2008; Laing, 2009).

The synchrony in complex networks is strongly affected by the topology of the networks, such as a regular lattice, random, small-world (SW), or scale-free (SF) structure, which lies as the foundation of diverse dynamics observed in naturally occurring complex networks, such as the central nervous system (Feldt et al., 2011). For instance, the hippocampal network during development follows a SF topology, and its hub nodes shapes synchronous activity in the network (Bonifazi et al., 2009). The developing cerebellum, in contrast, takes a regular connectivity, and its activity pattern is characterized by traveling waves (Watt et al., 2009). Theoretically, the effect of topology on synchrony has been studied extensively for single networks (Arenas et al., 2008; Rodrigues et al., 2016; Yamamoto et al., 2016). For example, in a single network of heterogeneous phase oscillators, the degree of phase synchrony increases as a regular lattice is rewired to SW and random networks (Hong et al., 2002). The effects of SW rewiring on networks has also been studied on integrate-and-fire neurons, and Netoff et al. (2004) investigated different types of synchronized activity depending on the rewiring probability and its relation to epileptic seizures. For SF networks, Lee (2005) used a system of phase oscillators and reported the dependence of the critical coupling strength for synchronization on the degree exponent. This leads us to hypothesize that the topology in subnetworks of NoNs would strongly influence their global dynamics.

In this study, we consider a simple NoN consisting of two coupled subnetworks and examine the effect of the topology of the subnetworks on the global synchrony. The topologies we consider are: random; SW; SF; and “super-hub,” which is characterized by the presence of a few hub nodes that are fully connected to all other nodes. Each node is represented as a Kuramoto phase oscillator, and the synchronization is evaluated by calculating the Kuramoto order parameter, *r*. We evaluate the networks in terms of the minimum coupling strength necessary to achieve synchronized state, by analyzing the minimum inter-node coupling strength necessary for *r* to exceed a threshold value. In the current study, the threshold was set to be *r* = 0.9. We show that in networks of heterogenous phase oscillators, the optimal topology varies between a single network and a NoN. Finally, we consider a NoN with three subnetworks and evaluate how coupling schemes between subnetworks of different mean frequencies affect the global synchrony.

## Materials and methods

### Network models

Each node in a network is modeled as a Kuramoto oscillator. The state of node *i* (*i* = 1, …, *N*) is described by its phase ϕ_*i*_, and its dynamics are calculated by the 4th-order Runge-Kutta method with a time step of *dt* = 0.05 s. Here, the time derivative of the phase, $\dot{\phi_{i}}$, is calculated as:

(1) $$\dot{\phi_{i}}=\omega_{i}-\frac{K}{2k}\sum_{j\in \Lambda_{i}}\sin\left(\phi_{i}-\phi_{j}\right),$$

where ω_*i*_ is the natural frequency, *K* is the coupling strength, *k* is the average node degree, and Λ_*i*_ is the set of nodes directly coupled to node *i*. ω_*i*_ is selected from a Gaussian distribution $N\left(\bar{\omega },\sigma_{\omega }^{2}\right)$ with a mean $\bar{\omega }$ of 4.5 and a standard deviation σ_ω_ of 0.15, and allocated randomly in the nodes, unless otherwise noted. The coupling strength is normalized using the average degree (Hong et al., 2002; Lee, 2005; Zhao et al., 2010), rather than the degree of individual nodes, in order to maintain the identity of high-degree nodes. Simulations are conducted for 250 and 500 s for the single network and NoN, respectively.

Each single network or a subnetwork of a NoN is composed of *N* = 50 nodes in most analyses, while networks with *N* = 1,000 nodes are employed to discuss the generalizability of the findings. In either case, the nodes are interconnected with undirected links with an average degree of six (*k* = 6). *K* is sampled at intervals of 0.05 and 0.1 for the single network and NoN, respectively. For each *K*, 250 networks with different sets of the adjacency matrix *A*_*ij*_, initial phase ϕ_*i*_(0), and natural frequency ω_*i*_ are sampled.

The network topologies that we consider are Erdös–Rényi random networks, Watts–Strogatz SW networks, Barabási–Albert SF networks, and a super-hub network. A random network is constructed by connecting randomly selected pairs of nodes until the necessary amount of connections ($\frac{N\times k}{2}$) are formed (Erdös and Rényi, 1960). A SW network is constructed by rewiring a one-dimensional lattice with a rewiring probability of 0.1 (Watts and Strogatz, 1998). A SF network is constructed as previously reported (Barabási and Albert, 1999), starting with seven fully-coupled nodes as an initial structure (21 connections) and sequentially adding 43 nodes with three connections per node under the preferential attachment rule, i.e., the probability of adding a new connection to an existing node *i* (1 ≤ *i* ≤ *N*′), Π(*k*_i_), is given by: $\Pi \left(k_{\text{i}}\right)=k_{\text{i}}/\overset{N^{\prime }}{\underset{j=1}{\sum }}k_{j}$, where *N*' is the number of existing nodes, and *k*_i_ the degree of *i*. A super-hub network comprises three hub nodes that are interconnected and also fully connected to other 47 nodes. Either zero or one connection is assigned between a given pair of nodes, except in the super-hub network, connections between hub nodes are set to three in order to retain constant the total number of connections in a network. Self-connections are not allowed in any of the networks.

### Analysis of synchrony

The degree of synchrony in the networks is evaluated using the order parameter, *r* (Hong et al., 2002; Arenas et al., 2008; Breakspear et al., 2010):

(2) $$r=\langle \left|\frac{1}{N}\sum_{j=1}^{N}e^{i\phi_{j}}\right|\rangle ,$$

where |⋯| and 〈⋯〉 denote the absolute values and time averages, respectively. In single networks, the first 50 s of the simulation are neglected, and the time average is obtained for the remaining 200 s. In the case of NoNs, the first 50 s after the coupling (250–300 s) are neglected, and the time average is obtained for the remaining 200 s (300–500 s). As discussed later, the time constants of transient phases are mostly in the order of seconds after which the order parameters of networks saturate. Hence the “burn-in” period of 50 s is sufficient to equilibrate the networks.

Analytically, the order parameter of a network is derived using the synchrony alignment function (Skardal et al., 2014), and is calculated using the natural frequency matrix and adjacency matrix *A* = [*A*_*ij*_]:

(3) $$J\left(\tilde{\omega },L\right)=\frac{1}{N}\sum_{j=2}^{N}\lambda_{j}^{-2}{\langle \nu^{j},\tilde{\omega }\rangle }^{2},$$

where $\tilde{\omega }$ is the normalized matrix of the natural frequency; *L* = [*L*_*ij*_] is the Laplacian matrix with *L*_*ij*_ = δ_*ij*_*k*_*i*_ − *A*_*ij*_ and $k_{i}=\overset{N}{\underset{j=1}{\sum }}A_{ij}$; λ_*j*_ is the *j*^th^ eigenvalue of *L*, ordered ascendingly; ν^*j*^ is the normalized eigenvector associated with λ_*j*_; and 〈·, ·〉 denotes the inner product. When a network is synchronizable, $J\left(\tilde{\omega },L\right)$ is approximately zero, and the analytical approximation of the order parameter can be calculated as:

(4) $$r\approx 1-\frac{J\left(\tilde{\omega },L\right)}{2{\left(K/2k\right)}^{2}}.$$

## Results and discussion

### Synchronization in single networks

We begin with a description of the basic properties of a single network of Kuramoto oscillators. A large portion of the results presented in this Section has already been under thorough investigation and are reviewed in Rodrigues et al. (2016), but is recapitulated here as a prolog to the next Section.

**Figure 1:**
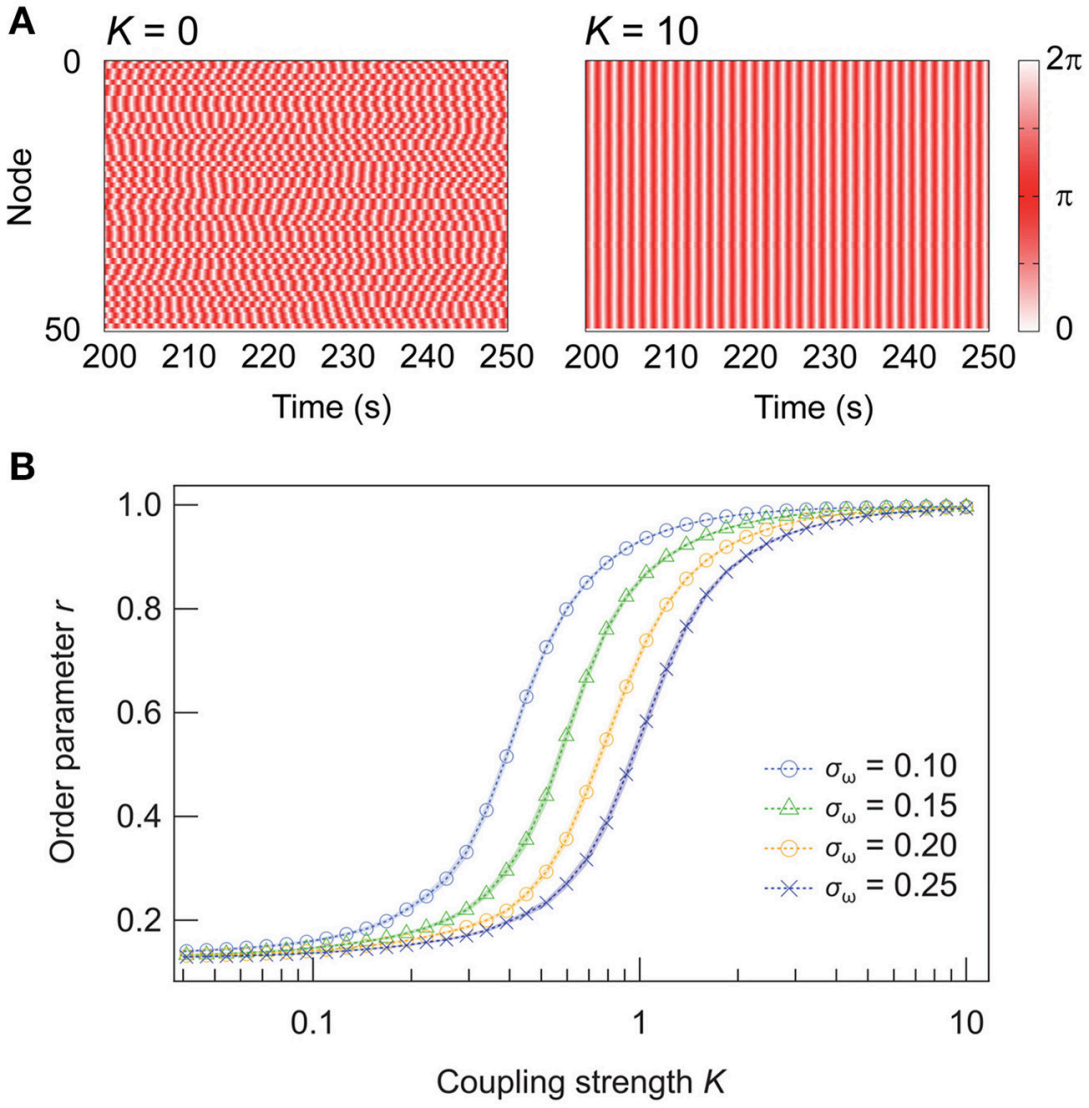
Synchronization in Kuramoto networks and the effect of the network inhomogeneity. **(A)** Dynamics of random networks ($\bar{\omega }$ = 4.5, σ_ω_ = 0.15) with coupling strengths of (a) *K* = 0 and (b) *K* = 10. **(B)** Dependence of the Kuramoto order parameter *r* on *K* for networks of inhomogeneous oscillators. A total of 250 networks are sampled for each condition, and their means are plotted. Shaded error bars represent 95% confidence intervals.

Figure 1A illustrates the dynamics of random networks without and with internode connections. Without any coupling (*K* = 0), the nodes oscillate independently at their natural frequencies, and the resulting order parameter for the network is *r* ≈ 0.1. In contrast, the nodes synchronize with sufficiently strong couplings (*K* = 10), yielding *r* ≈ 1. Figure 1B shows the dependence of *r* on *K*. When *K* reaches a critical value (*K* ≈ 0.2 for σ_ω_ = 0.15), *r* increases until it saturates at ~1. The effect of the inhomogeneity in the natural frequencies of the nodes is also shown in Figure 1B. Analytically, when the standard deviation σ_ω_ is scaled by a factor of *p, pK* is required to obtain an equivalent degree of synchrony (see Appendix). Hence, in the following discussion, we focus on a case where σ_ω_ = 0.15.

**Figure 2:**
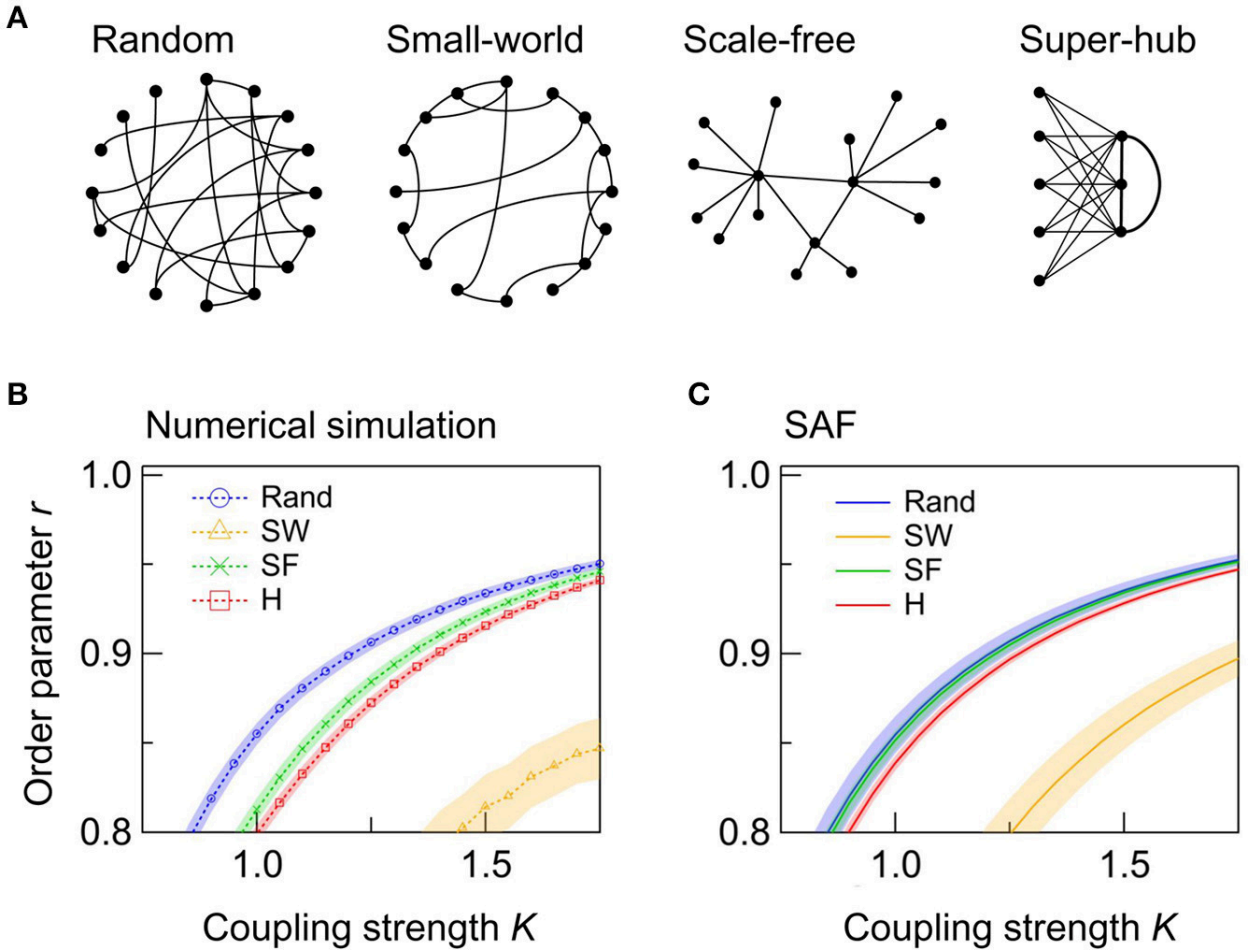
Effect of the network topology in single networks. **(A)** Schematic illustrations of the four types of network topologies considered in this study. For ease of viewing, the number of nodes *N* and their degrees *k* are varied. In the actual calculation, *N* and *k* are 50 and 6, respectively. **(B,C)** Dependence of *r* on *K* derived from **(B)** the numerical simulation and **(C)** the synchrony alignment function (SAF). The networks are random (Rand; blue circles), SW (orange triangles), SF (green crosses), and super-hub (H; red squares). A total of 250 networks are sampled for each condition, and their means are plotted. Shaded error bars represent 95% confidence intervals.

Next, we examine the effect of the network topology on the synchrony of a single network. We consider four types of topologies: random, SW, SF, and super-hub (Figure 2A). In all four networks, a synchronized state, which we define as *r* > 0.9, is achieved when *K* is sufficiently high. Figure 2B shows the dependence of *r* on *K* for networks with different topologies. Comparison of the random, SF, and super-hub networks reveals that random networks synchronize at the lowest *K* (Figure 2B). When the nodes are inhomogeneous, the variation in the natural frequencies of the hub nodes degrades the synchronization in the SF and super-hub networks. This trend is confirmed analytically from the evaluation of *r* from the synchrony alignment function (Figure 2C).

The SW networks always exhibit the lowest synchrony among the four topologies (Figures 2B,C). Importantly, the global synchronization in a network is not always a positive symptom. In the brain, for example, hypersynchronization of neurons is a phenotype of epilepsy (Netoff et al., 2004). In such cases, SW connectivity, such as that observed in the macroscopic wiring of the human cortex (Bullmore and Sporns, 2012), can be beneficial.

In summary, random connection is the most efficient strategy for synchronizing a network of inhomogeneous nodes with the minimum coupling strength.

### Synchronization in interconnected networks

**Figure 3:**
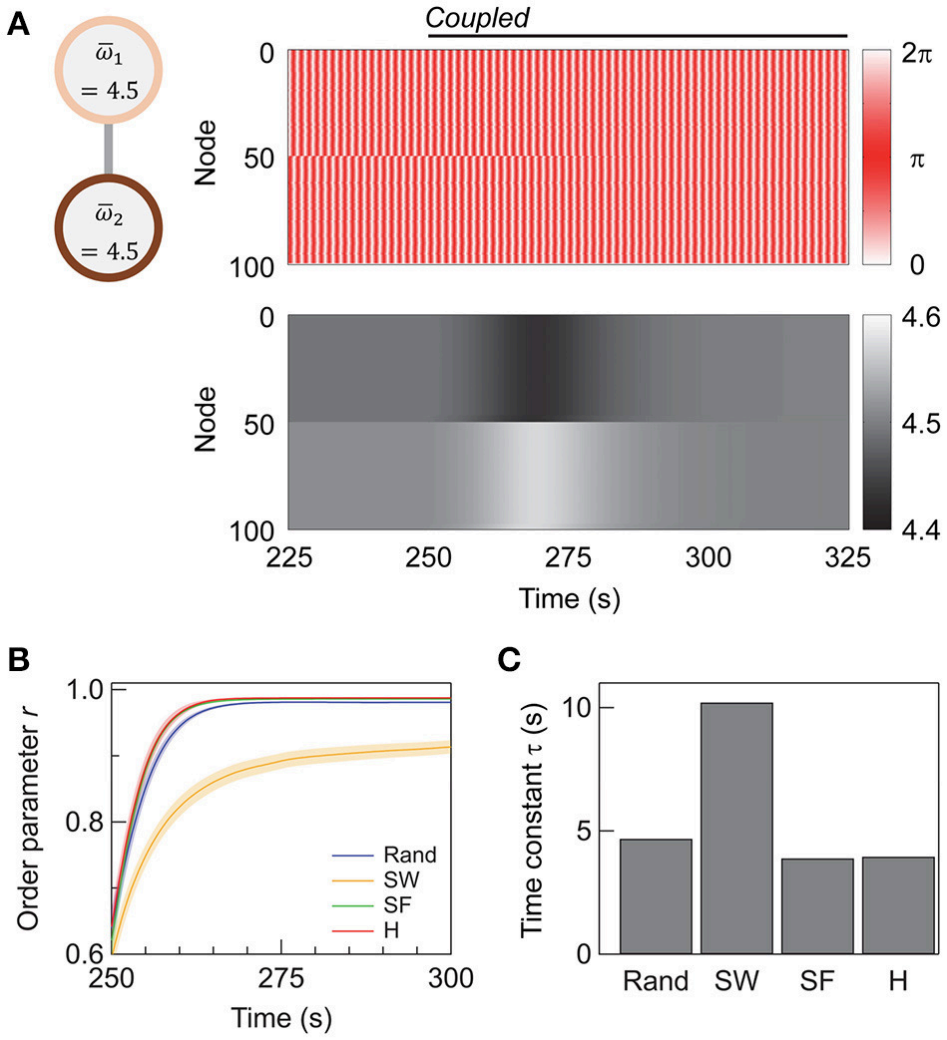
Synchronization in interconnected networks. **(A)** Schematic illustration of the interconnected network and the change in the node dynamics after the subnetworks are coupled at 250 s. The upper and lower panels show the evolution of phases and phase velocities, respectively. The topology of each subnetwork is super-hub with *N*_1_ = *N*_2_ = 50, $\bar{\omega }$ = 4.5, and *K*_intra_ = *K*_inter_ = 4. **(B)** Transient change in order parameter *r* upon the coupling of the subnetworks. A total of 250 networks are sampled and averaged for each topology. Shaded error bars represent 95% confidence intervals. The time average of *r* plotted here is calculated every 0.5 s instead of every 200 s. **(C)** The time constant τ of the transient change in *r* calculated for each topology. The abbreviations are as follows: R, random; SW, small-world; SF, scale-free; H, super-hub. The network parameters are *N*_1_ = *N*_2_ = 50, *K*_intra_ = 4, and *K*_inter_ = 10.

Next, we investigate the effect of the network topology of subnetworks in NoNs on the global synchrony. We consider the simplest case, i.e., a NoN consisting of two subnetworks (Figure 3A). Each subnetwork contains 50 nodes connected via the random, SW, SF, or super-hub strategy. Two subnetworks with an equivalent topology are coupled, with nine connections among three selected nodes. In coupling the subnetworks, nodes having the highest degree are selected from each subnetwork, since cortical networks are characterized by the rich-club organization (van den Heuvel and Sporns, 2013; Samu et al., 2014; Gal et al., 2017). This configuration is also inspired by previous studies on identical chaotic oscillators, which explored synchronization in NoNs comprised of two SF subnetworks and discovered that inter-subnetwork connections linking high-degree nodes allow efficient global synchrony (Zhao et al., 2011; Aguirre et al., 2014), while maintaining dynamical clustering of subnetworks (Zhao et al., 2011). For the super-hub topology, the three hub nodes are selected as connector nodes. The intra-subnetwork coupling strength, *K*_*intra*_, is kept constant at *K*_*intra*_ = 4, and the inter-subnetwork coupling strength, *K*_*inter*_, is varied.

Figure 3B shows the change in the order parameter averaged over the whole network, or the global order parameter, upon the addition of inter-subnetwork connections (*K*_*inter*_ = 10). Prior to the coupling of the two subnetworks, the nodes within each subnetwork are synchronized, as *K*_*intra*_ is sufficiently high (*K*_*intra*_ = 4). However, the synchronizing phases of the two subnetworks are independent, yielding a global order parameter of *r* ≈ 0.6. When the two subnetworks are coupled at *t* = 250 s, the global order parameter gradually increases, and the coupled subnetworks synchronize (*r* > 0.9). Note that although the network does not reach a fully synchronized state (*r* = 1), the whole network is in a steady state when *r* saturates (*t* > 300 s); this is confirmed by observing the distribution of the phase and phase velocity (Figure 3A). The steady state is hence different from the so-called “chimera state,” a state which coherent and incoherent domains coexist in (Laing, 2009) and appears as a transient phase in finite-sized Kuramoto networks (Wolfrum and Omel'chenko, 2011).

Analysis of the transient state after coupling the subnetworks (*t* > 250 s) reveals that the SF and super-hub networks have the fastest response. The results of curve fitting to an exponential function *r* = *r*_0_ − *A*exp[−(*t* − *t*_0_)/τ] are summarized in Table 1. The response to coupling the subnetworks is fastest in the SF (τ = 3.9 s) and super-hub (τ = 4.0 s) networks, followed by the random networks (τ = 4.7 s) (Figure 3C). For the SW network, the time constant τ is 10.2 s.

**Table 1:** Transient change in the order parameter after the coupling of two subnetworks.

	**Random**	**Small-world**	**Scale-free**	**Super-hub**
*r*_0_	0.98	0.93	0.99	0.99
*A*	0.36	0.30	0.38	0.36
τ (s)	4.7	10.2	3.9	4.0

*The results of the numerical simulation are fitted to an exponential function (r = r_0_ − A exp[−(t − t_0_)/τ]) to derive the parameters r_0_, A, and τ*.

**Figure 4:**
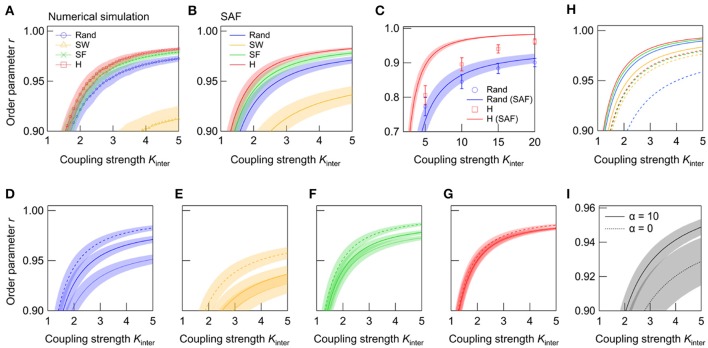
Dependence of *r* on *K*_inter_ in interconnected networks. **(A)** is derived from the numerical simulation, and **(B)** from the evaluation of the synchrony alignment function (SAF). The network topologies are random (blue), SW (green), SF (orange), and super-hub (red), with *N*_1_ = *N*_2_ = 50 and *K*_intra_ = 4. **(C)** *r*-*K*_inter_ relationships in large networks (*N*_1_ = *N*_2_ = 1000, *K*_intra_ = 4) with random (blue) and super-hub (red) subnetworks. Plots and solid lines represent the results obtained from the numerical simulation and SAF, respectively. **(D–G)** Effect of frequency allocation on synchrony in NoNs with **(D)** random, **(E)** SW, **(F)** SF, and **(G)** super-hub subnetworks (*N*_1_ = *N*_2_ = 50, *K*_intra_ = 4). Natural frequencies are reallocated so that outlying frequencies are placed either at high-degree hub nodes (broken lines) or at low-degree nodes (dotted lines). Solid line represents the default, random allocation. **(H)** Effect of connector node degree on synchrony. Nodes used to connect two subnetworks are chosen from either the highest-degree (solid lines) or lowest-degree (broken lines) nodes. Natural frequencies are allocated randomly, and *K*_intra_ was set to 12. The same color schemes are used for different topologies as in **(B)**. Error bars are removed to aid visualization. **(I)** Effect of rich-club organization on synchrony of hierarchically modular networks (*N*_1_ = *N*_2_ = 50, *K*_intra_ = 4, *k* = 6, *p*_in_ = 0.97). NoNs bearing subnetworks with rich-club organization (α = 10) is compared against those without it (α = 0). The two subnetworks are connected through the highest-degree nodes. A total of 250 networks are sampled for each condition, and their means are plotted. Shaded error bars represent 95% confidence intervals.

We next analyze *r* in the steady state. As shown in Figure 4, the *r* of NoN with random, SW, SF, and super-hub subnetworks increases with *K*_*inter*_. Despite the fact that the random topology synchronized (*r* > 0.9) at lowest coupling strength in the single networks, the super-hub topology is the most effective for synchronizing NoNs (Figure 4A). The result of the numerical simulation is fully supported by the analytical evaluation of order parameters from the synchrony alignment function (Figure 4B).

Since previous works have shown that the number of nodes in a network can dramatically impact the degree of synchrony in modular networks (Oh et al., 2005), we further study the effect of the subnetwork topology in a larger NoN consisting of 2,000 nodes. It is found that the advantage of the super-hub topology over random topology becomes more prominent when the network size is enlarged (Figure 4C). Note that the discrepancy between the synchrony alignment function and the numerical simulation, which is especially noticeable in the super-hub network is due to the extended relaxation time required for the networks to synchronize in the large networks.

One of the major advantage of the synchrony alignment function is the ability to combine the network structure with the allocation of natural frequencies. The effect of frequency allocation on the synchrony of NoNs (*N*_1_ = *N*_2_ = 50) with different subnetwork topologies is shown in Figures 4D–G. Here the natural frequencies, ω_*i*_, are reallocated depending on the node degree, *k*_i_: ω_*i*_ is sorted so that $\left|\omega_{i}-\bar{\omega }\right|$ either ascends or descends with *k*_i_. In the former case, outlying frequencies are allocated at the hub nodes, the first three of which are the connector nodes of the NoN. Contrarily, in the latter case, $\omega_{i}\approx \bar{\omega }$ for the hub nodes, and the outlying frequencies are allocated at the low-degree nodes. Analyses reveal that, in all subnetwork topologies, synchrony increases when outlying frequencies are allocated at the hubs (connector nodes). The direct interaction of outlying frequencies averages ω_*i*_ and allows them to oscillate at near $\bar{\omega }$. In contrast, when the outliers are placed at low-degree nodes, the cancelation does not occur and a group of nodes that oscillate away from $\bar{\omega }$ is formed, resulting in the degradation of global synchrony. In support of this, the effect of frequency reallocation is most effective in the random and SW networks, whereas the effect is minor in the super-hub and SF network, in which all peripheral nodes are under direct or strong control of the hubs.

We also investigate how the choice of connector nodes influences synchrony in NoNs of various subnetwork topologies. Here the synchrony alignment function is used to analyze *r* for NoNs constructed by connecting the lowest-degree nodes, instead of the highest-degree nodes, in two subnetworks. Natural frequencies are allocated randomly, and *K*_*intra*_ is increased to 12. The order parameters of the four topologies are summarized in Figure 4H, together with the results for the default case in which highest-degree nodes are used as connector nodes. The results show that the super-hub and SF topologies achieve highest synchrony even when the lowest-degree nodes are the connector nodes. Under this connection strategy, random networks exhibit the lowest synchrony, which is primarily due to a stochastic existence of nodes with very few intra-subnetwork connections. Such nodes can appear in SF networks as well, but in SF networks, the node is likely to be directly coupled to a high-degree node, and hence the global synchrony does not degrade even when the low-degree node is used as a connector.

The properties of the network topologies explored in the current study is not mutually exclusive. For instance, in the neural networks of the brain, SW and SF properties coexist as a result of cost-efficiency trade-off (Chen et al., 2013). In contrast, Watts-Strogatz SW network models generally lack SF properties, and the Barabási-Albert SF network models lack SW properties. In an attempt to model subnetworks bearing a topology that more closely resembles the real neural system, we further consider a hierarchically modular network with a rich-club organization (Zhou et al., 2006; Meunier et al., 2010; Wu et al., 2012; van den Heuvel and Sporns, 2013; Samu et al., 2014; Hilgetag and Goulas, 2016; Zamora-López et al., 2016; Gal et al., 2017). Such a subnetwork is generated by interconnecting two sub-subnetworks (each with *N* = 25 nodes and *p*_*in*_ × *N* × *k*/2 intra-connections) with (1 − *p*_in_) × *N* × *k*/2 inter-connections, where *p*_in_ designates the probability of intra-connections. The two nodes to be connected are chosen with a probability Π_*i*_ = $k_{i}^{\alpha }/\underset{j}{\sum }k_{j}^{\alpha }$, where *k*_i_ is the degree of node *i*, and α a parameter that tunes the preference of high-degree nodes. Finally, two of the subnetworks are connected through the top three highest-degree nodes. Comparison of *r* calculated from adjacency matrices generated with α = 0 (random selection) and α = 10 (rich-club selection) is summarized in Figure 4I. In hierarchically modular networks, the higher synchrony is achieved by coupling the sub-subnetworks through high-degree hub nodes. Together with the previous result (Figure 4H), the result underscores the importance of hubs in considering synchrony within NoNs.

The variation in the effective topology for synchronizing a single network and NoN is the primary finding of the present work. In a single network, the random topology is more robust to node inhomogeneity (section Synchronization in Single Networks). However, in NoNs, the existence of high-degree hub nodes and connection of the subnetworks through the hubs is effective for achieving synchronization. This advantage overwhelms the aforementioned disadvantage in individual networks, and hence, super-hub and SF networks synchronize with weaker coupling strength than random networks. The result is consistent with a recent report on the synchronization in multilayer networks of identical chaotic oscillators, in which synchrony was achieved at weaker inter-layer coupling strength when a network with layers configured under the SF topology rather than random topology (Leyva et al., 2017). Analytical and numerical studies on the NoNs (or multilayer networks) with non-identical subnetwork topology is an important topic (Um et al., 2011; Leyva et al., 2017), which awaits future research.

### Coupling networks of different frequencies

In real systems, the natural frequencies of the nodes are often distributed. In the previous sections, we considered this by using nonzero values for σ_ω_. Previously, a number of reports have investigated effective connection strategies for synchronizing an isolated single network comprised of inhomogeneous oscillators (Gleiser and Zanette, 2006; Brede, 2008). Going up one hierarchy in scale, it is reasonable to consider a case in which the subnetworks in a NoN have distinct average natural frequencies ${\bar{\omega }}_{\alpha }$. In the last section of this paper, we explore an optimal wiring strategy for synchronizing such a network.

**Figure 5:**
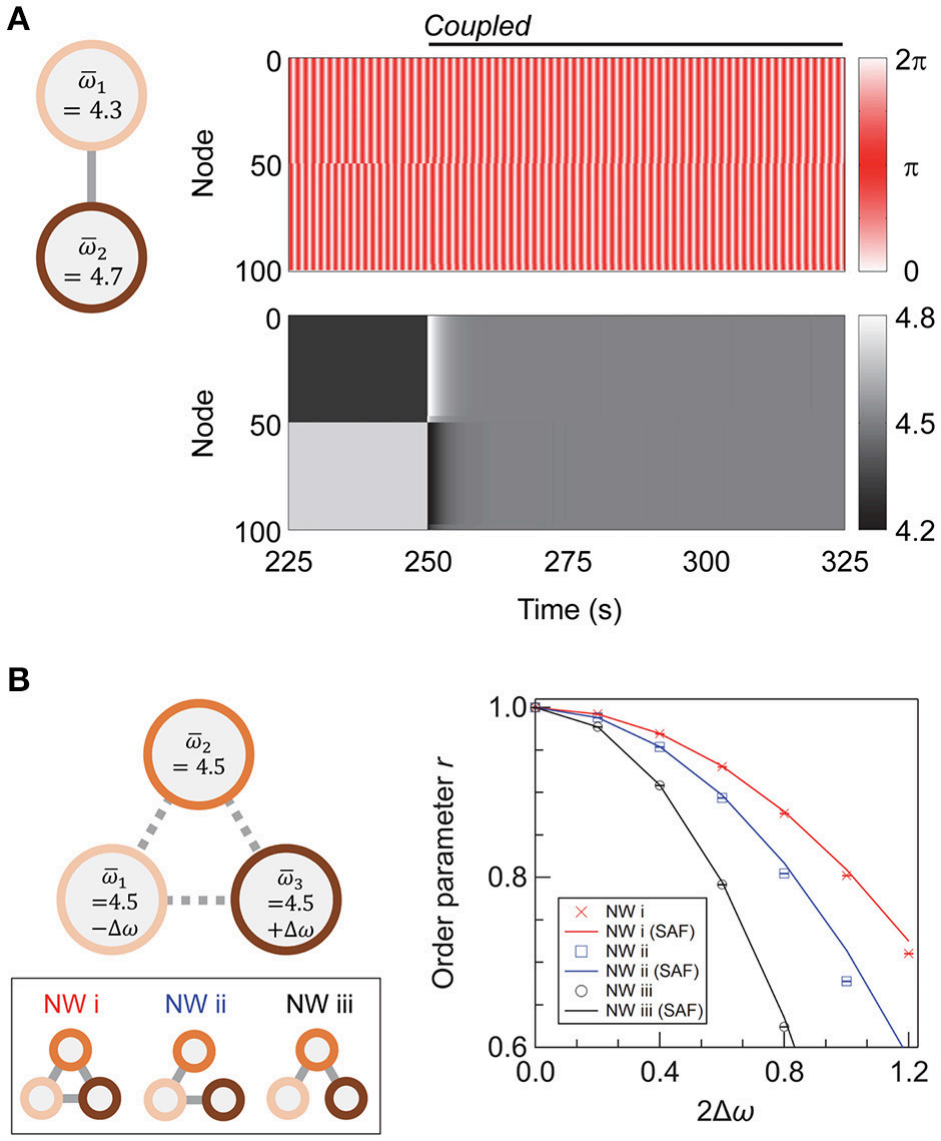
Coupling networks of different frequencies. **(A)** A schematic showing the networks under consideration, and the evolution of phases (upper panel) and phase velocities (lower panel) of the nodes. The average natural frequencies of the two subnetworks are ${\bar{\omega }}_{1}=4.3$ and ${\bar{\omega }}_{2}=4.7$. **(B)** A schematic showing the three-network system and the effect of inter-network coupling on synchronization. A total of 250 networks are sampled for each condition, and their means are plotted. Error bars representing 95% confidence intervals are depicted but are invisible. The network topology of the individual networks is super-hub, with *N*_1_ = *N*_2_ (= *N*_3_) = 50, σ_ω_ = 0, *K*_intra_ = 4, and *K*_inter_ = 40.

When two subnetworks with different average natural frequencies, ${\bar{\omega }}_{1}$ and ${\bar{\omega }}_{2}$, are coupled with a sufficiently high coupling strength, they synchronize at a mean frequency of $\left({\bar{\omega }}_{1}\;+\;{\bar{\omega }}_{2}\right)/2$, as shown previously in Li et al. (2008) and Wu et al. (2012). Figure 5A shows the transient dynamics of coupled super-hub networks with ${\bar{\omega }}_{1}=4.3$ and ${\bar{\omega }}_{2}=4.7$. After inter-subnetwork coupling at *t* = 250 s, the two networks begin to interact, and the oscillating frequencies of their nodes gradually converge to 4.5.

We further investigate whether there is an efficient coupling scheme for a NoN comprising three subnetworks of different ${\bar{\omega }}_{\alpha }$. We assume that each subnetwork retains the super-hub topology with *N*_α_ = 50 (α = 1, 2, 3), *k* = 6, *K*_intra_ = 4, and *K*_inter_ = 40 and that the total number of inter-subnetwork links is nine. The average natural frequencies of the three subnetworks are ${\bar{\omega }}_{1}=\omega_{0}-\Delta \omega$, ${\bar{\omega }}_{2}=\omega_{0}$, and ${\bar{\omega }}_{3}=\omega_{0}+\Delta \omega$, with ω_0_ = 4.5. To simplify the discussion, the variation of the natural frequency within each subnetwork (σ_ω1_, σ_ω2_, σ_ω3_) is set as zero. A total of 250 realizations are sampled for each condition.

Three types of inter-subnetwork connections are considered (Figure 5B). We denote the NoN with full coupling as Network (i), which serves as a reference in the subsequent discussion. In Networks (ii) and (iii), only two pairs of subnetworks are coupled. Subnetworks 1 and 2 are coupled in both networks, and in Network (ii), two subnetworks with a large discrepancy—Subnetworks 1 and 3—are coupled, whereas in Network (iii), two subnetworks with a small difference—Subnetworks 2 and 3—are coupled. A network with two couplings of Subnetworks 1 and 3 and Subnetworks 2 and 3 is mathematically equivalent to Network (ii).

The order parameter for Networks (i)–(iii) and its dependence on Δω is shown in Figure 5B. Network (i), with fully coupled subnetworks, has the highest degree of synchrony for all values of Δω. When the wiring resource is limited to coupling two pairs of subnetworks, the direct coupling of subnetworks with a dissimilar average frequency [Network (ii)], is favorable for achieving a high global synchrony in the steady state.

The mechanism of Network (ii) being more synchronizable than Network (iii) can be understood from the previous discussion regarding the steady-state frequency of the coupled subnetworks (Figure 5A). Considering that two coupled subnetworks synchronize at their mean frequency, Network (ii) can be coarse-grained into a set of interacting subnetwork-pairs with the average natural frequency of $4.5-\frac{\Delta \omega }{2}$ (constituted by the interaction of Subnetworks 1 and 2) and 4.5 (Subnetworks 1 and 3). For Network (iii), the frequencies are $4.5-\frac{\Delta \omega }{2}$ (Subnetworks 1 and 2) and $4.5+\frac{\Delta \omega }{2}$ (Subnetworks 2 and 3). The delta in these frequencies is smaller for Network (ii) than for Network (iii); hence, Network (ii) can withstand a larger deviation in the natural frequencies. However, the shift in the *r*-Δω relationship with the connection strategy is nonlinear, and the value of Δω for realizing a certain *r* in Network (ii) is not equal to 2 × Δω for Network (iii).

In summary, the direct coupling of dissimilar subnetworks is favorable for achieving a high degree of global synchrony with a limited number of connections.

## Conclusion

We investigated the effect of the subnetwork topology on the synchronization of interconnected networks, or NoNs. Although random connection was favorable for synchronizing individual networks, the SF and super-hub topology with high-degree hub nodes exhibited highest synchrony in NoNs. This variation in the optimal topology in single and interconnected networks is the major finding of our study. The use of a phase oscillator model as local node dynamics allowed us to analytically investigate the structure-function relationships in NoNs via the synchrony alignment function. The results provide a first-order approximation to the study of dynamics within complex networks of biologically more plausible neural oscillators, such as the neural mass models (Zhao et al., 2010, 2011; Zamora-López et al., 2016). Indeed, the collective dynamics of a Kuramoto oscillator system has been shown to resemble that of neural mass models (Hoppensteadt and Izhikevich, 1997; Zamora-López et al., 2016), which highlights the neuroscientific relevance of the phase reduction approach (Breakspear et al., 2010; Rodrigues et al., 2016). The directionality of the connections and interaction delays are the factors that we also simplified or neglected in this study. The former is critical for analyzing the propagating signals in networks, and the latter has been shown to be able to settle interconnected networks at an atypical global oscillatory state (Louzada et al., 2013).

In the brain, synchronized neural activity is critical for its function at various spatial resolutions. At the microscopic scale, it modulates the synaptic weights (Kubota and Kitajima, 2008; Benchenane et al., 2011) through spike-timing dependent plasticity, whereas at the macroscopic scale, it supports efficient signal transfer between distant brain regions (Senkowski et al., 2008; Deco and Kringelbach, 2016). Excessive synchrony, however, in a wide area is pathophysiological, and is a phenotype of neurological disorders such as epilepsy (Netoff et al., 2004; Truccolo et al., 2014). In this line, the current work provides insights into how the topology of subnetworks contributes to balance synchrony and asynchrony in complex networks comprised of interacting subsystems. In particular, our results suggest that coexistence of SF and SW properties, which promote and suppress synchrony, respectively, would facilitate networks to achieve this balance.

The balance of synchrony and asynchrony, and the resulting complexity of the dynamics, has been quantified based on, e.g., the probability distribution of pairwise correlation between nodes (Zhao et al., 2010; Zamora-López et al., 2016), the probability distribution of joint states of nodes (Marshall et al., 2016), modularity analysis on the correlation matrix (Zhou et al., 2006; Zhao et al., 2011), and Granger causality analysis on the temporal pattern of joint states (Shanahan, 2008). Such measures have been applied not only to analyze synthetic networks but also anatomical brain connectomes, which revealed that the dynamical complexity is maximized therein (Zhao et al., 2010; Zamora-López et al., 2016). Most of the measures are applicable to phase oscillator systems, and their combination would provide a theoretical framework for further investigating the structure-function relationships within NoNs, such as brain networks.

## Author contributions

HY, SK, and MN: Conceived and designed the research; HY and FS: Performed the simulations and analyzed the results; AH-I and MN: Supervised the research; HY and SK: Wrote the manuscript; FS, AH-I, and MN: Reviewed and edited the manuscript.

### Conflict of interest statement

The authors declare that the research was conducted in the absence of any commercial or financial relationships that could be construed as a potential conflict of interest.

## Appendix

We show here that the order parameter, *r* [Equation (2)], of a Kuramoto oscillator network with a natural frequency distribution $\omega_{i}\sim N\left(\bar{\omega },\sigma_{\omega }^{2}\right)$ and a coupling strength *K* does not change when both the σ_ω_ and *K* are simultaneously multiplied by and arbitrary value *p* ∈ ℝ, i.e., σ_ω_ and *K* are replaced such that σ_ω_ → *pσ*_ω_ and *K* → *pK*. If we define a new phase variable, ψ_*i*_, as $\psi_{i}\equiv \phi_{i}-\bar{\omega }t$, then Equation (1) can be rewritten as:

(A1) $$\dot{\psi_{i}}\omega_{i}^{\prime }-\frac{K}{2k}\sum_{j\in \Lambda_{i}}\sin\left(\psi_{i}-\psi_{j}\right),$$

where $\omega_{i}^{\prime }\equiv \omega_{i}\;-\;\bar{\omega }$ obeys the distribution of $\omega_{i}^{\prime }\sim N(0,\sigma_{\omega }^{2})$. Since $r=\frac{1}{N}\langle \left|\sum_{j=1}^{N}e^{i\phi_{j}}\right|\rangle =\frac{1}{N}\langle \left|\sum_{j=1}^{N}e^{i\left(\psi_{j}+\bar{\omega }t\right)}\right|\rangle =\frac{1}{N}\langle \left|\sum_{j=1}^{N}e^{i\psi_{j}}\right|\rangle$, the order parameter for ϕ_*i*_ is equal to that for ψ_*i*_, indicating that:

(A2) $$r\left(N\left(\bar{\omega },\sigma_{\omega }^{2}\right);K\right)=r\left(N\left(0,\sigma_{\omega }^{2}\right);K\right),$$

where, for the simplicity of notation, we denoted the order parameter *r* for the system with ω_*i*_~$N\left(\bar{\omega },\sigma_{\omega }^{2}\right)$ and *K* as $r\left(N\left(\bar{\omega },\sigma_{\omega }^{2}\right);K\right)$.

Next, we consider a rescaling of time *t* by defining $t^{\prime }\equiv \frac{t}{p}$. Using the new time variable *t*', the dynamics of ϕ_*i*_ under *t'* is:

(A3) $$\frac{d\phi_{i}}{dt^{\prime }}=p\frac{d\phi_{i}}{dt}=p\omega_{i}-\frac{pK}{2k}\sum_{j\in \Lambda_{i}}\sin\left(\phi_{i}-\phi_{j}\right).$$

Since rescaling of time does not influence a temporally averaged value, *r* for the two systems in Equations (1) and (A3) are equal. And hence, the following relationship is derived:

(A4) $$r\left(N\left(0,{\left(p\sigma_{\omega }\right)}^{2}\right);pK\right)=r\left(N\left(0,\sigma_{\omega }^{2}\right);K\right).$$

Finally, by using the relationships in Equations (A2) and (A4), we obtain:

(A5) $$r\left(N\left(\bar{\omega },{\left(p\sigma_{\omega }\right)}^{2}\right);pK\right)=r\left(N\left(\bar{\omega },\sigma_{\omega }^{2}\right);K\right),$$

meaning that the replacement of σ_ω_ → *p*σ_ω_ and *K* → *pK* does not affect the value of *r*.
